# Single Antiplatelet Therapy With Ticagrelor in Flow-Diversion Treatment of Ruptured Dissecting Cerebral Pseudoaneurysms: A Meta-Analysis

**DOI:** 10.7759/cureus.106088

**Published:** 2026-03-29

**Authors:** Saeed A Alqahtani, Saad AlQahatani

**Affiliations:** 1 Neurological Surgery, MedStar Georgetown University Hospital, Washington, DC, USA; 2 Neurosurgery, Houston Methodist Hospital, Houston, USA

**Keywords:** flow diversion, flow-diverter embolization, pseudoaneurysm rupture, safety and efficacy, single antiplatelet therapy, subarachnoid hemorrhage, ticagrelor

## Abstract

Background and aim: The management of ruptured dissecting cerebral pseudoaneurysms (RDPs) remains a significant neurosurgical challenge. Flow-diversion devices (FDDs) have emerged as an effective therapeutic option; however, their use typically necessitates dual antiplatelet therapy (DAPT), which carries an inherent risk of hemorrhagic complications. This concern has led to growing interest in single antiplatelet therapy (SAPT) with ticagrelor, given its more predictable pharmacodynamic profile. Nevertheless, the evidence supporting the use of ticagrelor-based SAPT in this setting remains limited. This study evaluated the efficacy and safety of single antiplatelet therapy (SAPT) with ticagrelor among patients following flow-diversion treatment for ruptured cerebral pseudoaneurysms (RDPs).

Methods: In this study, the Preferred Reporting Items for Systematic Reviews and Meta-Analyses (PRISMA) guidelines were followed for screening and selecting research articles. Electronic databases used for data extraction were PubMed, ClinicalTrials.gov, and Cochrane Library. The research timeline was set from January 2015 to September 2024. The Newcastle-Ottawa Scale (NOS) was applied to assess the quality of observational studies. This study used the Review Manager (RevMan) software version 5.4 (London, England: Cochrane Collaboration) for pooled analysis.

Results: Through a pooled analysis of six cohort or observational studies involving 1118 patients, the findings indicate that single antiplatelet therapy (SAPT) with ticagrelor may be comparable to standard dual antiplatelet therapy in managing thrombosis risk. The pooled analysis showed that the composite outcome of thromboembolic events, major hemorrhagic complications (OR: 0.86, 95% CI: 0.53-1.38), and hemorrhagic complications alone (OR: 0.58, 95% CI: 0.21-1.62) were not significantly different between patients receiving single antiplatelet therapy (SAPT) with ticagrelor and those receiving control antiplatelet regimens (including aspirin and clopidogrel), although the point estimates favored ticagrelor-based SAPT. Similarly, no statistically significant differences were observed in thromboembolic events (OR: 1.15, 95% CI: 0.57-2.32) or mortality (RR: 1.17, 95% CI: 0.21-6.39) between the treatment and control groups.

Conclusion: Overall, this meta-analysis suggests that single antiplatelet therapy (SAPT) with ticagrelor may be a feasible and potentially safe alternative to dual antiplatelet therapy in patients undergoing flow-diverter treatment for ruptured dissecting cerebral pseudoaneurysms (RDPs). The available evidence indicates comparable safety profiles, although the certainty of these findings remains limited. Therefore, well-designed randomized controlled trials and prospective comparative studies are warranted to more definitively evaluate efficacy, safety, and optimal patient selection.

## Introduction

In neurosurgery, the management of ruptured dissecting cerebral pseudoaneurysms (RDPs) becomes complex and a clinical challenge due to the high risk of rebleeding [1,2]. Pseudoaneurysms are localized enlargements in the walls of cerebral arteries that develop after injury, trauma, infections, or connective tissue disorders to the vessel layers [3]. These abnormal expansions of cerebral arteries cause subarachnoid hemorrhage (SAH) and other severe neurological sequelae, in turn leading to high mortality rates due to aneurysms [4]. For the management of these complex lesions, flow-diverting devices (FDDs) have emerged as an effective therapeutic strategy among advanced endovascular techniques [5]. These flow-diverting devices improve blood flow dynamics through stents having high metal surface area coverage, subsequently allowing the reconstruction of the parent artery and treating the aneurysm thrombosis [6,7].

In recent years, flow-diversion stents such as the pipeline embolization device (PED) have gained much importance due to their clinical outcomes in treating complex aneurysms [8]. However, integration of flow-diversion stents for endovascular treatment requires antiplatelet therapy to prevent thromboembolic complications [9,10]. Management of ruptured cerebral pseudoaneurysms emerged as a critical clinical challenge due to complexities related to timely diagnosis and treatment of outcomes. Pseudoaneurysms exhibit reserved radiographic features requiring rapid and accurate diagnosis [11]. Acute hemorrhage frequently requires surgical intervention; nevertheless, the risks of surgery, such as infection, rebleeding, and neurological impairments, can result in unfavorable outcomes. The care is made more difficult by elements like the patient's comorbidities and the pseudoaneurysm's anatomical location. In these complicated circumstances, a multidisciplinary approach is necessary to maximize results.

Dual antiplatelet therapy (DAPT) using aspirin and clopidogrel following flow-diverter placement has been applied as a standard therapeutic strategy for managing stent thrombosis risk [12,13]. Moreover, DAPT resulted in an increased risk of adverse events such as hemorrhagic complications. Due to these concerns, researchers and clinicians have emphasized the implications of single antiplatelet therapy (SAPT) as a safer endovascular treatment strategy [14]. Among various antiplatelet drugs, ticagrelor emerged as a potent P2Y12 inhibitor in single antiplatelet therapy (SAPT) for the management of ruptured aneurysms, which provides more clinical outcomes and predictable pharmacodynamics in comparison to clopidogrel [15].

In cardiovascular studies, ticagrelor, compared to clopidogrel, proved effective in platelet inhibition, extending its investigation in neurovascular management [16]. Its dynamic binding method inhibits the formation of platelets more consistently, potentially making it a safer option for patients necessitating emergency aneurysm treatment [17]. Recent research indicates that ticagrelor, when taken as a single antiplatelet drug, could mitigate both risks of thrombosis and bleeding in patients receiving flow-diversions for ruptured cerebral pseudoaneurysms [18]. However, evidence on the effectiveness of ticagrelor as monotherapy in this scenario is limited, necessitating a thorough review to determine its safety and efficacy [19].

The number of randomized controlled trials (RCTs) evaluating the outcomes of antiplatelet strategies for flow-diversion devices used to treat ruptured pseudoaneurysms is limited [20,21]. Several studies on DAPT's outcomes in flow-diversion treatment for unruptured aneurysms have been published previously [22]. However, there is a lack of studies evaluating the outcomes of SAPT in emergent situations involving rupture. In retrospective case series and cohort studies, single antiplatelet treatment (SAPT) with ticagrelor has shown promise due to its excellent pharmacokinetic profile and potential for minimizing hemorrhagic events [23]. However, the risks associated with insufficient platelet inhibition versus over-inhibition and clinical guidelines related to single antiplatelet treatment (SAPT) with ticagrelor remain unclear, which demands further investigation [10,24]. Therefore, this study evaluated the efficacy and safety of single antiplatelet therapy (SAPT) with ticagrelor among patients following flow-diversion treatment for ruptured dissecting cerebral pseudoaneurysms (RDPs). Through pooled analysis of multiple cohort and observational studies, this study would clarify several clinical outcomes, including thromboembolic events, hemorrhagic complications, and mortality rates.

## Materials and methods

Study design

In this study, the Preferred Reporting Items for Systematic Reviews and Meta-Analyses (PRISMA) guidelines were followed for the screening and selection of research articles [25]. Our study was a meta-analysis of already published cohort and observational studies, so additional ethical review was not required.

Search strategy

The research articles related to the study's aim, "single antiplatelet therapy with ticagrelor in flow-diverter treatment for ruptured dissecting cerebral pseudoaneurysms,” were extracted from various databases using the PRISMA guidelines [25,26]. Electronic databases used for data extraction were PubMed, ClinicalTrials.gov, and Cochrane Library. The MeSH keywords used for data extraction were ("Single Antiplatelet Therapy" OR "SAPT" OR “ticagrelor”) AND ("Flow Diverter Treatment” OR “flow diversion”) AND ("thromboembolic events" OR “TEE” OR "Rate of thrombosis" OR "hemorrhagic complications"). The research timeline was set from January 2015 to September 2024.

Selection criteria

The predefined selection criteria helped screen research articles in this study. Only those articles in this study met the following criteria: (1) studies involving patients diagnosed with aneurysms or ruptured dissecting cerebral pseudoaneurysms, (2) studies have shown that patient populations receive flow-diverter treatment, (3) studies discuss the outcomes related to thromboembolic events, hemorrhagic complications, and mortality rates, (4) studies based on randomized controlled trials (RCTs) and observational studies, and (5) studies published in English and with full text.

Only those studies were excluded as follows: (1) studies involving populations with other neurovascular disorders, (2) studies evaluating other interventions rather than antiplatelet therapy, (3) already published systematic reviews, meta-analyses, scoping reviews, and case studies, and (4) studies that were published in other languages rather than English, and with non-full-text papers or duplicated publications.

Data extraction

For each included paper, we extracted the demographic information related to authors, year of study, country, study population, sample size, study design, and primary outcomes such as outcomes related to composite (thromboembolic event {TEE} or major hemorrhagic event), thromboembolic events, hemorrhagic complications, one-point modified Rankin Scale (mRS) worsening (mRS is used to measure the degree of disability in patients who have had a stroke), and mortality rates from included articles as mentioned in Table 1 [27-34].

**Table 1:** Characteristics of included studies. mRS: modified Rankin Scale

Studies	Country	Study population	Study design	Study follow-up	Dose of intervention	Composite (thromboembolic events or major hemorrhagic events)	Thromboembolic events (TEE)	Hemorrhagic complications	One-point mRS worsening	Mortality rates
Papaxanthos et al. (2023) [29]	France	260 patients (mean age: 58 years); 104 in the treatment group, 156 in the control group	Multicentric retrospective study	6 months	T: ticagrelor loading dose (180 mg); P: clopidogrel loading dose (150 mg)	T: 12; P: 17	T: 11; P: 14	T: 1; P: 3	T: 7; P: 13	T: 3; P: 4
Mohammaden et al. (2020) [30]	Egypt	24 patients (mean age: 47.7 years)	Retrospective study	6 months	T: ticagrelor loading dose (180 mg); P: control	-	T: 2; P: 0	T: 1; P: 0	-	-
Tanburoglu and Andic (2021) [31]	Turkey	6 patients; 1 in the treatment group, 5 in the control group (prasugrel)	Retrospective study	3 months	T: ticagrelor 180-mg loading dose; P: 60 mg loading dose	-	-	T: 1; P: 0	-	T: 1; P: 0
Park et al. (2021) [32]	Canada	236 patients (mean age: 57.5 years); 135 in treatment, 101 in the control group	Single-center cohort study	6 months	T: ticagrelor loading dose (180 mg); P: clopidogrel loading dose (150 mg)	T: 7; P: 10	T: 4; P: 4	T: 6; P: 13	-	T: 1; P: 3
Moore et al. (2017) [33]	USA	103 patients; 53 in treatment (ticagrelor) group, 50 in the control group	Retrospective cohort study	6 months	T: ticagrelor loading dose (180 mg); P: clopidogrel loading dose (150 mg)	-	-	T: 3; P: 6	-	-
May et al. (2024) [34]	USA	570 patients; 126 in treatment, 360 in control group	Retrospective cohort study	6 months	T: ticagrelor loading dose (180 mg); P: clopidogrel loading dose (150 mg)	T: 10; P: 30	-	-	-	-

Quality assessment

The quality of the included studies was evaluated using proper tools based on the study design. The Newcastle-Ottawa Scale (NOS) was applied for the quality assessment of observational studies [27]. A score of >7 for included studies was considered low risk, scores of 5-7 for included studies indicated moderate risk, and scores of <5 for included studies showed high risk. Any disagreement in risk of bias assessment was resolved through consensus.

Statistical analysis

The Review Manager (RevMan) software version 5.4 (London, England: Cochrane Collaboration) was used for pooled analysis in this study [28]. The analysis was done to evaluate the odds ratio (OR) and risk ratio (RR) of expected outcomes after SAPT with ticagrelor. Moreover, heterogeneity was assessed using the Q test and I^2^ statistic. A significant difference was considered if p>0.05. The magnitude of heterogeneity was quantified by I^2^ statistics (mild: 0-30%; moderate: 31-50%; high: >50%). Funnel-pooled estimates were reported to determine publication bias.

## Results

Search results

**Figure 1:**
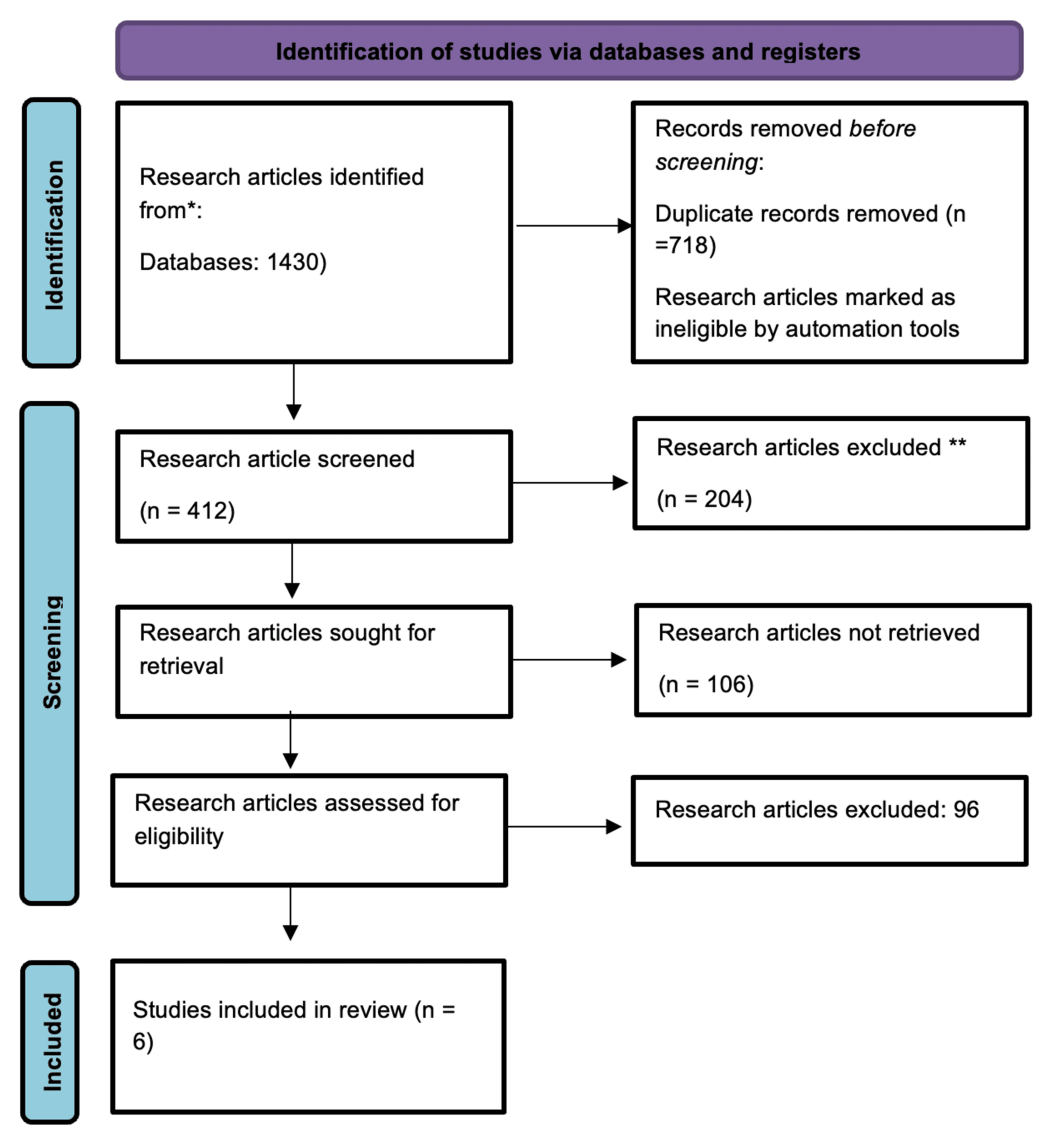
Flow chart of PRISMA guidelines for screening and selection of research studies. *No data available. PRISMA: Preferred Reporting Items for Systematic Reviews and Meta-Analyses

By following PRISMA guidelines in the screening and selecting research articles, approximately 1430 studies were identified from the three electronic databases mentioned above to fulfill the research aims of this meta-analysis-based study. The primary screening was performed on 412 research articles, of which 117 were excluded. About 208 research articles were retrieved before applying the eligibility criteria. The eligibility criteria were applied to 102 research papers, and only six studies met the eligibility criteria (Figure 1).

Description of included studies

The characteristics of all included studies were summarized in Table 1. All included studies involved patients (50-80 years old) with cerebral aneurysms and pseudoaneurysms following flow-diverter treatment. A total of 1118 patients from six included studies were analyzed in this study. All patients in intervention groups received single antiplatelet therapy (SAPT) with ticagrelor in comparison to placebo or other drugs such as clopidogrel. To acess heterogeneity in this study, one study from France [29], one from Egypt [30], one from Turkey [31], one from Canada [32], and two studies from the United States were included [33,34]. The median duration of follow-up was six months.

Quality assessment of included studies

Among the six included studies, two were high risk [30,32], two were moderate risk [29,34], and two were low risk, as assessed by the NOS checklist (Table 2) [31,33].

**Table 2:** Quality assessment of included studies. *No data available.

Studies	Selection				Comparability		Outcome			Total
	Representative of the exposed cohort	Selection of external control	Ascertainment of exposure	Outcome of interest not present	Main factor	Additional factor	Assessment of outcome	Sufficient follow-up time	Adequacy of follow-up time	
Papaxanthos et al. (2023) [29]	*	0	*	0	*	0	*	*	*	6/9
Mohammaden et al. (2020) [30]	*	*	*	0	*	0	*	0	0	5/9
Tanburoglu and Andic (2021) [31]	*	*	0	*	*	*	*	0	*	7/9
Park et al. (2021) [32]	*	*	*	0	*	0	*	0	0	5/9
Moore et al. (2017) [33]	*	*	0	*	*	*	*	8	*	8/9
May et al. (2024) [34]	*	0	*	0	*	0	*	*	*	6/9

Primary outcomes

Composite (Thromboembolic Events or Major Hemorrhagic Events)

**Figure 2:**
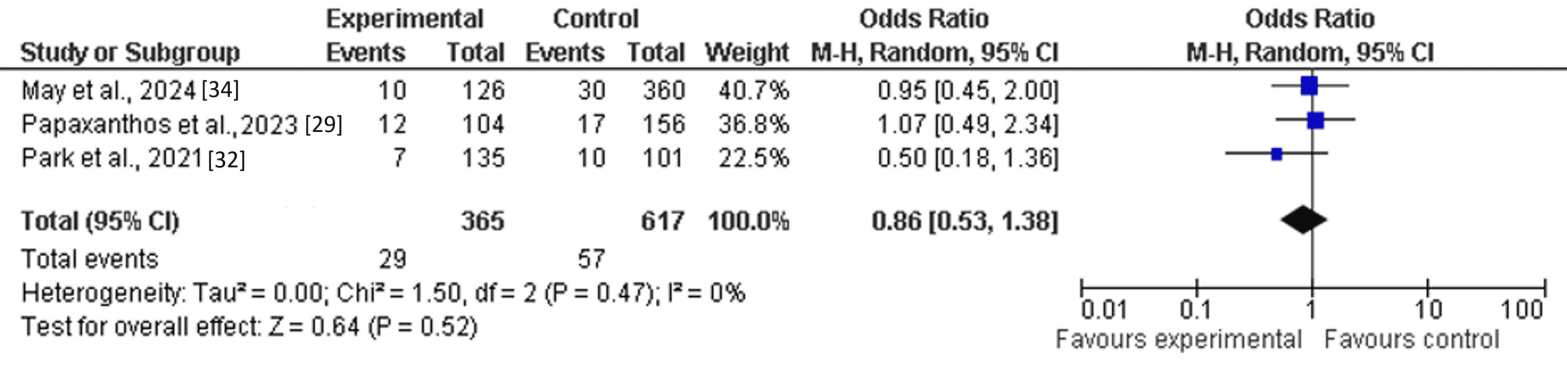
Forest plot of composite events among the treatment group as compared to placebo.

Among the six included studies, three reported a composite outcome of thromboembolic events and major hemorrhagic complications in patients receiving single antiplatelet therapy (SAPT) with ticagrelor. Pooled analysis demonstrated no statistically significant difference between the ticagrelor-based SAPT group and the control group (other antiplatelet regimens, including clopidogrel) (OR: 0.86; 95% CI: 0.53-1.38; Z=0.64). There was no evidence of heterogeneity across studies (I²=0%; df=2) (Figure 2).

Thromboembolic Events (TEE)

**Figure 3:**
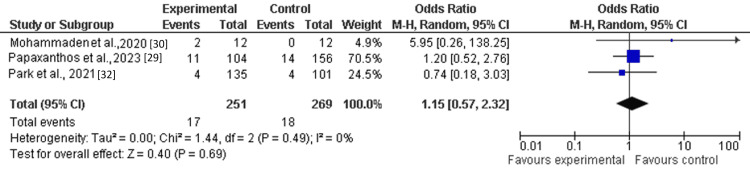
Forest plot of thromboembolic events (TEEs) among the treatment group as compared to placebo.

Only three out of six included studies reported the thromboembolic events (TEEs) as a clinical outcome of single antiplatelet therapy (SAPT) with ticagrelor among patients with ruptured dissecting cerebral pseudoaneurysms. The pooled analysis showed that thromboembolic events (TEEs) were not significantly different between the treatment group (SAPT with ticagrelor) and the control group (OR: 1.15; 95% CI: 0.57-2.32) (Figure 3).

Hemorrhagic Complications

**Figure 4:**
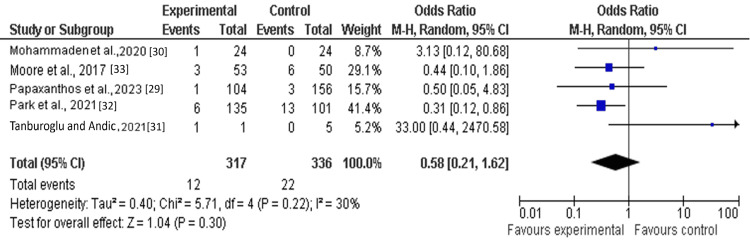
Forest plot of hemorrhagic complications among the treatment group as compared to placebo.

Among the six included studies, three reported hemorrhagic complications as a clinical outcome in patients receiving single antiplatelet therapy (SAPT) with ticagrelor. The pooled analysis demonstrated no statistically significant difference between the ticagrelor-based SAPT group and the control group (other antiplatelet regimens, including clopidogrel) (OR: 0.58; 95% CI: 0.21-1.62; Z=1.04). Moderate heterogeneity was observed across studies (I²=30%; df=4) (Figure 4).

Mortality Rates

**Figure 5:**
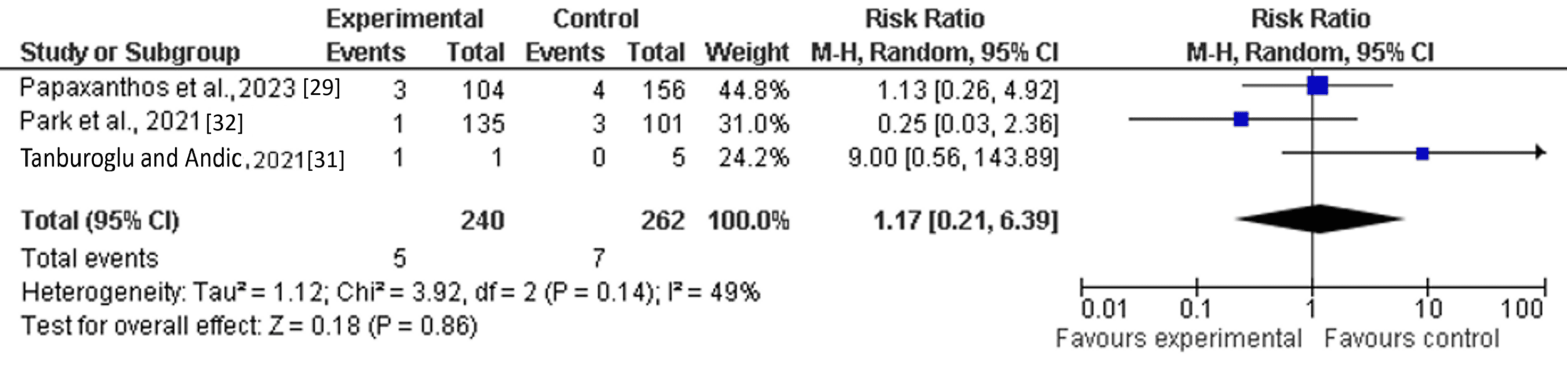
Forest plot of the risk ratio of mortalities among the treatment and placebo groups.

Among the six included studies, three reported mortality as a clinical outcome in patients receiving single antiplatelet therapy (SAPT) with ticagrelor. The pooled analysis demonstrated no statistically significant difference in mortality between the ticagrelor-based SAPT group and the control group (other antiplatelet regimens, including clopidogrel) (RR: 1.17; 95% CI: 0.21-6.39; Z=0.18). Moderate heterogeneity was observed across studies (I²=49%; df=2) (Figure 5).

## Discussion

This study aimed to evaluate the efficacy and safety of single antiplatelet therapy (SAPT) with ticagrelor among patients following flow-diversion treatment for ruptured dissecting cerebral pseudoaneurysms (RDPs), comparing it to different treatments using a meta-analysis research approach. Through pooled analysis of six cohort or observational studies involving 1118 patients, the findings indicate that single antiplatelet therapy (SAPT) with ticagrelor may be comparable to standard dual antiplatelet therapy in managing thrombosis risk. The findings reported that an odds ratio of composite (thromboembolic events or major hemorrhagic event) (OR: 0.86; 95% Cl: 0.53-1.38) and hemorrhagic complications (OR: 0.58; 95% Cl: 0.21-1.62) favored the treatment group (SAPT with ticagrelor) as compared to the control (other antiplatelet medications such as clopidogrel). However, the pooled analysis demonstrated that thromboembolic events (TEE) (OR: 1.15; 95% CI: 0.57-2.32) and mortality (RR: 1.17; 95% CI: 0.21-6.39) were not significantly different between the treatment group (SAPT with ticagrelor) and the control group [29-34].

The treatment of ruptured dissecting cerebral pseudoaneurysms (RDPs) remains a significant issue in neurosurgery and interventions in neurosurgery. The development of flow diversion has provided a promising, less-invasive treatment option for these complex vascular disorders [35,36]. However, the best antiplatelet therapy to promote these strategies remains debated. The findings of this study reported that single antiplatelet therapy with ticagrelor may be a sustainable option in the management of ruptured dissecting cerebral pseudoaneurysms (RDPs). Ticagrelor, a potent P2Y12 receptor antagonist, proved effective in providing rapid action and robust clinical outcomes due to a favorable pharmacokinetic profile compared with other antiplatelet agents, such as clopidogrel [37,38]. Its use has been associated with reduced thromboembolic events across several vascular treatments, making it a desirable choice for patients undergoing flow-diversion procedures [39,40].

Ticagrelor effectively improves platelet function through its reversible binding to the P2Y12 receptor and is needed after surgical intervention [41]. This aspect is especially crucial in flow-diversions, where the risk of acute problems such as stent thrombosis is significant. Our findings show that using ticagrelor as a single antiplatelet drug was comparable to the standard dual antiplatelet therapy [42]. Furthermore, a decrease in bleeding problems resulting from single antiplatelet medication should not be underestimated. Several trials included in this meta-analysis found that patients taking ticagrelor had fewer hemorrhagic incidents than those on dual antiplatelet treatment [43]. This result is critical, considering the already high risk of hemorrhage caused by ruptured dissecting cerebral pseudoaneurysms (RDPs). The relationship between preventing thrombotic events and reducing the risk of bleeding is vulnerable, and ticagrelor appears to reach a better balance.

Ticagrelor has emerged as a critical option for the management of patients with acute coronary syndrome (ACS) through inhibition of the P2Y12 receptor. The advantage of ticagrelor is its quick action with platelet inhibition, which occurs within hours of administration. In contrast, clopidogrel exhibited a delayed effect because it requires metabolic activation, unlike ticagrelor [44]. In acute clinical settings, the rapid action of ticagrelor prioritizes its implications when patients need timely intervention. There is rapid recovery of platelet function due to the quick binding of ticagrelor to the P2Y12 receptor, particularly when urgent surgery or intervention is required [45]. Wallentin et al. reported a reduction in major cardiovascular events, including stroke and heart attack, after the administration of ticagrelor as compared to clopidogrel through the Platelet inhibition and patient Outcomes (PLATO) trial [46]. It is also a suitable option for people with stents because it has been linked to decreased stent thrombosis [47]. With its quick absorption and roughly 7-h half-life, ticagrelor's pharmacokinetic profile is advantageous and makes it possible to use a practical 180 mg loading dosage followed by 90 mg twice daily. Crucially, ticagrelor's actions are independent of hepatic metabolism, which results in more uniform antiplatelet activity over patient groups [48].

However, there are some potential adverse events of ticagrelor along with enormous advantages, such as dyspnea, bradycardia, and bleeding, that emphasize effective monitoring among high-risk patients [49]. Medication interactions should also be evaluated to maximize patient safety, especially with drugs that affect CYP3A4. Ticagrelor offers improved efficacy and safety for individuals at risk of thrombotic events, making it an essential development in antiplatelet therapy overall.

Recent research has examined the implementation of single antiplatelet therapy (SAPT) with flow-diversions (FDs) to treat cerebral aneurysms, specifically ruptured ones [50]. SAPT with ticagrelor has demonstrated encouraging benefits, with reduced thromboembolic complication rates compared to aspirin alone [51]. Hydrophilic-coated FDs under SAPT have shown promise for treating ruptured aneurysms, with few device-related complications. One trial found that FDs with peri-interventional followed by prasugrel monotherapy were effective for treating ruptured blood blisters and dissecting aneurysms [10,52]. While SAPT seems to have a good safety profile, particularly with ADP-receptor inhibitors, the overall bleeding complication rate was modest at 0.1%. However, more extensive cohort studies are required to assess further SAPT outcomes in FD therapies [24,53].

Our meta-analysis has potential limitations, with enormous advantages that have impacted the validity of the results in this study. Firstly, the number of included studies was limited, following observational, retrospective, or prospective study designs with high publication bias and low heterogeneity. Secondly, the outcome and complication rates varied across studies due to differences in antiplatelet medication regimens, aneurysm status, SAPT dosage, and duration. Furthermore, the antiplatelet regimen regarding duration, dosage, and patient characteristics has not been standardized. However, these findings may offer valuable insight for future research.

However, it is essential to highlight that the available research on ticagrelor's use in this specific scenario is limited, with only a few trials focusing on its effectiveness in flow-diversion therapies for ruptured dissecting cerebral pseudoaneurysms (RDPs) [54,55]. This meta-analysis emphasizes the importance of more extensive, multicenter randomized controlled studies that confirm the findings. The inherent heterogeneity in patient demographics, study methods, and outcome measures among the included studies may induce biases that limit the applicability of the results [56]. Accordingly, robust evidence from well-designed randomized controlled trials and prospective comparative studies is needed to more definitively establish the efficacy and safety of ticagrelor-based SAPT, as well as to refine patient selection and optimize antiplatelet strategies in this high-risk population.

## Conclusions

This meta-analysis suggests that ticagrelor-based single antiplatelet therapy (SAPT) may be a feasible and potentially safe alternative to dual antiplatelet therapy in patients undergoing flow-diverter treatment for ruptured dissecting cerebral pseudoaneurysms (RDPs). The pooled data demonstrate comparable rates of thromboembolic and hemorrhagic complications between treatment strategies, supporting consideration of SAPT in carefully selected cases where bleeding risk is a concern. However, these findings are limited by the observational design of the included studies, variability in treatment protocols, and small sample sizes. Prospective, randomized studies are needed to more definitively establish the safety, efficacy, and optimal application of ticagrelor-based SAPT in this high-risk population.
